# Clinical outcomes of treatment with locking compression plates for distal femoral fractures in a retrospective cohort

**DOI:** 10.1186/s13018-019-1401-9

**Published:** 2019-11-26

**Authors:** Masahiro Kiyono, Tomoyuki Noda, Hiroshi Nagano, Takashi Maehara, Yasuaki Yamakawa, Yusuke Mochizuki, Takahiko Uchino, Suguru Yokoo, Koji Demiya, Kenta Saiga, Yasunori Shimamura, Toshifumi Ozaki

**Affiliations:** 1 Department of Orthopedic Surgery, Graduate School of Medicine, Dentistry and Pharmaceutical Sciences, Okayama University, 2-5-1, Shikata-cho, Kitaku, Okayama City, Okayama Prefecture 700-8558 Japan; 2 Department of Musculoskeletal Traumatology, Graduate School of Medicine, Dentistry and Pharmaceutical Sciences, Okayama University, 2-5-1 Shikata-cho, Okayama City, Okayama, 700-8558 Japan; 3 Department of Orthopedic Surgery, Kagawa Prefectural Central Hospital, 1-2-1 Asahi-Machi, Takamatsu City, Kagawa Prefecture 760-8557 Japan; 4 Department of Orthopedic Surgery, Kagawa Rosai Hospital, Jotocho, Marugame, Kagawa Prefecture 763-8502 Japan; 5 Department of Community and Emergency Medicine, Graduate School of Medicine, Dentistry and Pharmaceutical Sciences, Okayama University, 2-5-1, Shikata-cho, Kitaku, Okayama City, Okayama Prefecture 700-8558 Japan

**Keywords:** Distal femur fracture, Relative stability, Bridging plate, Locking compression plate, Empty hole

## Abstract

**Background:**

Plate fixation is one of the standard surgical treatments for distal femoral fractures. There are few reports on the relationship between the screw position and bone union when fixing by the bridging plate (relative stability) method.

**Methods:**

This retrospective study included 71 distal femoral fractures of 70 patients who were treated with the locking compression plate for distal femur (DePuy Synthes Co., Ltd, New Brunswick, CA, USA). The following measurements were evaluated and analyzed: (1) bone union rate, (2) bridge span length (distance between screws across the fracture), (3) plate span ratio (plate length/bone fracture length), (4) number of empty holes (number of screw holes not inserted around the fracture), and (5) medial fracture distance (bone fracture distance on the medial side of the distal femur). Patient demographics (age), comorbidities (smoking, diabetes, chronic steroid use, dialysis), and injury characteristics (AO type, open fracture, infection) were obtained for all participants. Univariate analysis was performed on them.

**Results:**

Of 71 fractures, 26 fractures were simple fractures, 45 fractures were comminuted fractures, and 7 fractures resulted in non-union. Non-union rate was significantly higher in comminuted fractures with bone medial fracture distance exceeding 5 mm.

Non-union was founded in simple fractures with bone medial fracture distance exceeding 2 mm, but not significant (*p* = 0.06). In cases with simple fractures, one non-union case had one empty hole and one non-union case had four empty holes, whereas in cases with comminuted fractures, five non-union cases had two more empty holes.

**Conclusions:**

We concluded that bone fragment distance between fracture fragments is more important than bridge span length of the fracture site and the number of empty holes. Smoking and medial fracture distance are prognostic risk factors of nonunion in distal femoral fractures treated with LCP as bridging plate.

## Introduction

Distal femoral fractures comprise only 0.4% of all fractures and 4–6% of femoral fractures [1, 2]. However, the non-union rate of distal femoral fractures lies between 0 and 34%, indicating considerable variation [3, 4]. Surgical treatment can either be retrograde intramedullary nail fixation or be plate fixation, with plate fixation having a wide indication for various fractures types [5, 6].

Regarding plate fixation, basic fixation is generally recommended to achieve absolute stability using lag screws in simple fractures; however, lag screw fixation cannot be performed in transverse fractures [7]. Moreover, it is impossible to achieve absolute stability with rigid internal fixation in comminuted fractures. In such cases, it is necessary to use a locking plate as a bridging plate to fix the fracture site [8]. The optimal insertion holes for the screws on the proximal side of the locking plate (when used as a bridge) are currently debated [9, 10]. Therefore, in this study, we examined the correlation between bone union and screw position, as the X-ray parameter.

## Patients and methods

This study received Institutional Review Board approval, and patients provided oral and written informed consents. The present study was approved by Okayama University Graduate School of Medicine, Dentistry, and Pharmaceutical Sciences and Okayama University Hospital (Ethics Committee No. 1712-035).

This study included 71 distal femoral fractures in 70 patients (23 males, 47 females; mean age, 68.0 years [range, 16–91 years]) who were treated using the locking compression plate for distal femur (DePuy Synthes Co., Ltd, New Brunswick, CA, USA) at a university hospital and related facilities between April 2005 and December 2015. One female patient had bilateral femoral fractures. Cases in which the proximal and distal bone fragments were fixed using a lag screw and those in which the medial plate was used during the initial surgery were excluded. All surgery was performed by surgeons with more than 15 years of orthopedic experience at university hospital and related facilities.

The mean follow-up period was 20.8 months (range, 5–33 months) (Table 1). Simple fractures were defined as 33A2 and 33C1 classifications and comminuted fractures were classified as 33A3, 33C2, and 33C3 according to the Arbeitsgemeinschaft für Osteosynthesefragen/Orthopedic Trauma Association (AO/OTA) fracture classification [11]; we excluded 33B type AO/OTA fractures. There were 26 simple fractures and 45 comminuted fractures based on plain antero-posterior (AP) and lateral X-ray images (Table 2).

**Table 1:** Characteristics of distal femoral fractures

Characteristics	Number of fractures
Sex	
Male	23
Female	47*
AO/OTA fracture classification	
33A	38
A2	21
A3	17
33C	33
C1	5
C2	13
C3	15
Open or closed	
Open	19
Closed	52

*AO/OTA* Arbeitsgemeinschaft für Osteosynthesefragen/Orthopedic Trauma Association

*One female had bilateral distal femoral fractures

**Table 2:** Characteristics of non-unions compared with fractures that healed

Characteristics	Non-union (*n* = 7)	Union (*n* = 64)	*p* value
Age, years	61.5 ± 18.4	68.4 ± 18.7	0.25
Open fracture	4	15	0.07
MIPO	6	55	0.67
Simple fracture (AO/OTA 33A2/C1)	2	24	0.49
Comminuted fracture (AO/OTA 33A3/C2/C3)	5	40	
Number of empty holes	2.0 ± 0.8	1.67 ± 1.89	0.17
Bridge span length (mm)	78.3 ± 29.5	85.9 ± 30.0	0.68

Data are presented as mean ± standard deviation or number. Comparisons were conducted using Fisher’s exact test

*MIPO* minimally invasive plate osteosynthesis, *AO/OTA* Arbeitsgemeinschaft für Osteosynthesefragen/Orthopedic Trauma Association

In this study, we investigated (1) bone union rate, (2) bridge span length (distance between screws across the fracture), (3) plate span ratio (plate length/bone fracture length), (4) number of empty holes (number of screw holes not inserted around the fracture), and (5) medial fracture distance (bone fracture distance on the medial side of the distal femur).

**Fig. 1:**
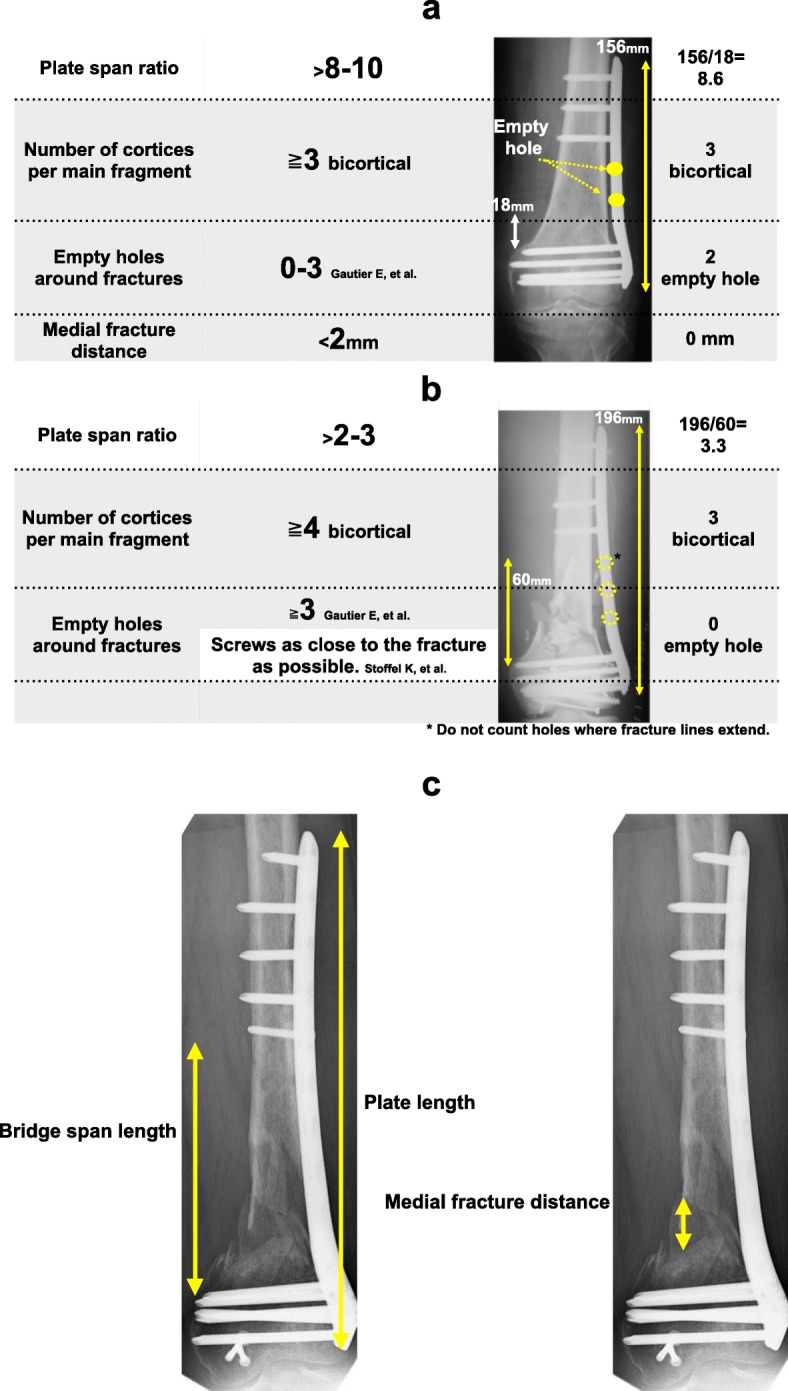
Conclusive rules by Gautier et al. [14] and Stoffel et al. [15]. **a** Conclusive rules of simple fracture for 33A2 and 33C1. **b** Conclusive rules of comminuted fracture for 33A3, 33C2, and 33C3. **c** The definition of the part measured this time

Bone union was defined as three out of four instances of cortical bridging on AP and lateral radiographs [12]. Non-union was defined as a state in which bone union was not achieved within 4 months after the initial surgery. Working length of a plate was defined as the distance between the first screws on either side of the fracture [13]. Plate span ratio was measured with reference to the length of the plate relative to the length of the fracture line, as proposed by Gautier et al. and Stoffel et al. [14, 15]. Guidelines for defining the terms “plate span ratio,” “empty hole,” and “medial fracture distance” are provided in Fig. 1a, b, and c. Measurements of the medial fracture distance between the proximal fragment and the medial cortex of the distal fragment were based on plain AP and lateral X-ray images. For cases in whom the bone fracture site was shortened by surgeon, the medial distance was regarded as 0 mm. When an intermediate bone fragment exceeding 20 mm was included in the measurement range, the distance between the both ends and the main bone fragment was measured.

Fisher’s exact test was performed for statistical comparisons of open fractures, minimally invasive plate osteosynthesis, plate span ratio, and medial fracture distance between those with bone union (bone union group) and those with non-union (non-union group). The Mann-Whitney *U* test was performed for statistical comparisons of the average number of empty holes and the average bridge span length. Results with a *p* value of < 0.05 were considered significant.

## Results

In this study, non-union was diagnosed in 7 of 71 cases. Non-union was diagnosed 2 of 26 (7.7%) cases with a simple fracture and 5 of 45 (11.1%) cases with a comminuted fracture (Table 2). As a salvage surgery for 7 non-union cases, two patients added a plate on the medial side of the femur (one case also used bone grafting), the other one performed only bone grafting, two cases left an existing plate with replacing the position of the screw, one case replaced with a long plate, and last case was shortened the fracture site and performed bone grafting and plate fixation. Fourteen of all comminuted fracture cases and bone union were treated through shortening the fracture site as Rekha reported [16], and non-union was not observed in these cases. Tables 2, 3, and 4 summarize the univariate analysis. Only smoking and medial fracture distance were statistically significant (*p* < 0.05) independent risk factors predictive of nonunion requiring intervention. Age, open fracture, MIPO, diabetes, steroid use, infection, AO classification, plate span ratio, and bridge span length were of no predictive value.

**Table 3:** Characteristics of simple fractures (OTA 33A2, C1) (*n* = 26)

Characteristics	Non-union (*n* = 2)	Union (*n* = 24)	*p* value
Age, years	76.2 ± 11.6	77.5 ± 3.5	0.89
Open fracture	0	1	0.92*
MIPO	1	22	0.22*
AO/OTA 33A2/C1	A2: 2	A2: 19; C1: 5	
Plate span ratio			
≥ 8–10	0	3	0.77*
< 8–10	2	21	
Number of empty holes	2.5 ± 1.5	1.58 ± 0.9	0.17^†^
0 holes	0	1	
1 hole	1	14	
≥ 2 holes	1	9	
Medial fracture distance			
≥ 2 mm	2	5	0.06*
< 2 mm	0	19	
Bridge span length (mm)	72.5 ± 27.5	72.2 ± 24.9	0.87^†^

Data are presented as mean ± standard deviation or number

*MIPO* minimally invasive plate osteosynthesis, *AO/OTA* Arbeitsgemeinschaft für Osteosynthesefragen/Orthopedic Trauma Association

*Fisher’s exact test

^†^Mann-Whitney *U* test

**Table 4:** Characteristics of comminuted fractures (OTA 33A3/C2/C3) (*n* = 45)

Characteristics	Non-union (*n* = 5)	Union (*n* = 40)	*p* value
Age, years	61.5 ± 18.0	68.4 ± 19.9	0.19
Open fracture	4	14	0.14*
MIPO	5	34	0.47*
AO/OTA 33A3/C2/C3	A3, 2; C2, 3	A3, 15; C2, 10; C3, 15	
Plate span ratio			
≥ 2–3	5	36	0.61*
< 2–3	0	4	
Number of empty holes	2.0 ± 0.5	1.2 ± 1.1	0.07^†^
0 holes	0	9	
1 hole	0	13	
≥ 2 holes	5	18	
Medial fracture distance‡			
≥ 5 mm	5	0	0.00*
< 5 mm	0	40	
Bridge span length (mm)	81.2 ± 30.7	92.5 ± 29.9	0.54^†^

Data are presented as mean ± standard deviation or number

*MIPO* minimally invasive plate osteosynthesis, *AO/OTA* Arbeitsgemeinschaft für Osteosynthesefragen/Orthopedic Trauma Association

*Fisher’s exact test

^†^Mann-Whitney *U* test

‡There was no significant difference at 2 mm; however, there was a significant difference when the case was divided at 5 mm

The mean bridge span length of simple fractures in the bone union and non-union groups was 72.2 mm (range, 25–110 mm) and 72.5 mm (range, 45–100 mm), respectively (Table 3). On the other hand, the mean bridge span length of comminuted fractures in the bone union and non-union groups was 92.5 mm (range, 45–190 mm) and 81.2 mm (range, 40–110 mm), respectively (Table 4). No statistically significant difference in the working lengths of the fracture site was observed between simple fractures and comminuted fractures in both groups (Tables 3 and 4).

With respect to plate span ratio, 3 of 24 (12.5%) cases with simple fractures in the bone union group fit the plate length that was > 8–10 times longer than the overall fracture length, whereas 21 of 24 (87.5%) cases did not. Additionally, none of the cases in the non-union group fit the plate length of > 8–10 times longer than the overall fracture length (Table 3). Furthermore, 36 of 40 (90%) cases with comminuted fractures in the bone union group fit the plate length that was > 2–3 times longer than the overall fracture length, whereas 4 of 40 (10%) cases did not. All the cases in the non-union group fit the plate length of > 2–3 times longer than the overall fracture length (Table 4).

Of the cases with simple fractures, there was one non-union case with one empty hole and one non-union case with four empty holes (Table 3). Conversely, in cases with comminuted fractures, there were five non-union cases with more two empty holes (Table 4).

Of 26 simple fractures, there were 7 cases with a medial fracture distance of ≥ 2 mm, and the non-union rate was 28.5% (2 of the 7 cases) (Table 3). Additionally, there were 13 comminuted fractures with a medial fracture distance of ≥ 2 mm, and the non-union rate was 30.7% (4 of the 13 cases). In cases with comminuted fractures, there were five cases with bone fracture distance of ≥ 5 mm (each length was 5, 5, 6, 8, 9 mm) on the medial side of the distal femur; all cases resulted in non-union, resulting in a 100% non-union rate in those with a wide bone distance.

Open fractures were observed in 19 of 71 (26.8%) cases (Table 1). Of the comminuted fractures, 18 open fractures were observed; four cases resulted in non-union, which was not a significantly larger proportion than that in the bone union group (Table 4).

**Fig. 2:**
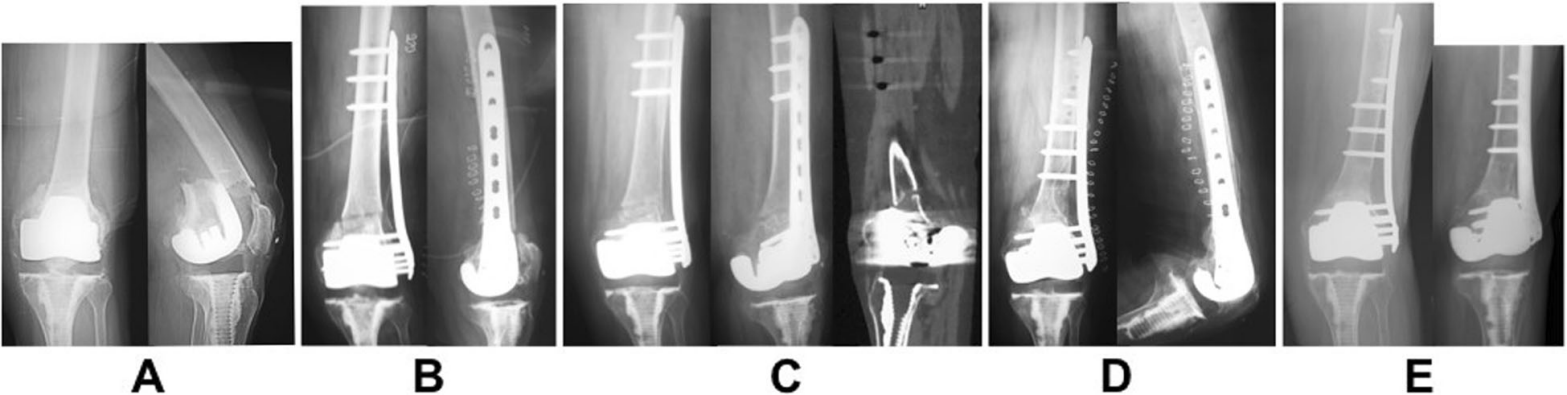
**a** Radiograph in a 74-year-old woman with total knee arthroplasty. **b** Postoperative radiograph. **c** Radiograph and CT showed no callus at 6 months postoperatively. **d** Screws were inserted near the fracture during re-operation. **e** Radiograph 1 year after re-operation

**Fig. 3:**
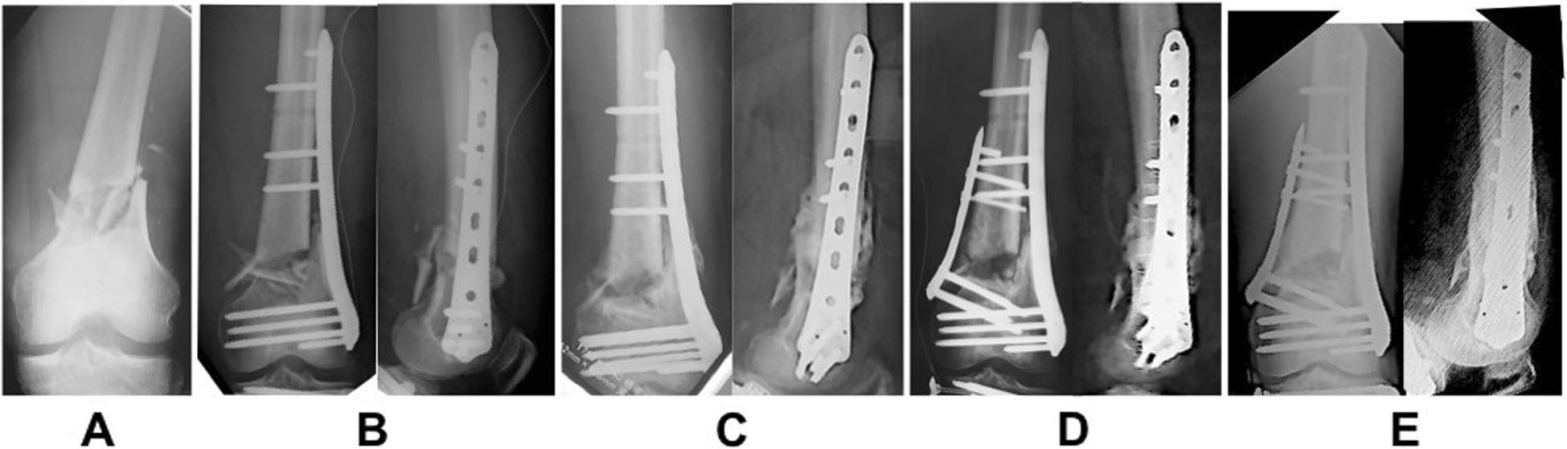
**a** Radiograph with Gustilo type-IIIA in a 73-year-old man. **b** Postoperative radiograph. **c** Radiograph and CT showed non-union 8 months postoperatively. **d** Another plate was added during re-operation to shorten the fracture. **e** Radiograph 1 year after re-operation

**Fig. 4:**
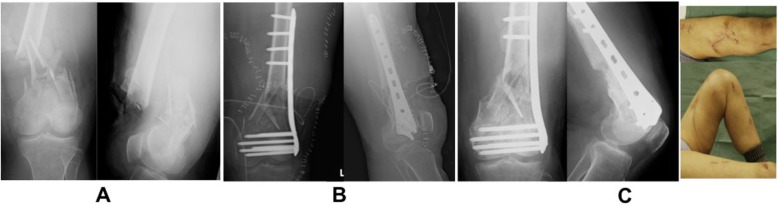
**a** Radiograph with Gustilo type-IIIA in an 80-year-old woman. **b** Postoperative radiograph. **c** Radiograph and CT showed callus formation 2 months postoperatively; left knee range of motion was 0–140°

Several example cases are shown in Figs. 2, 3, and 4. Case 1 was distal femur fracture in a 74-year-old woman with TKA after a fall. Her injury was a simple transverse fracture that resulted in non-union. The alignment was reduced and LCP-DF was used as a bridging plate. It showed that the plate length was 196 mm, fracture distance was 5 mm, plate span ratio was 196/5 = 39.2, bridge span length was 100 mm, medial fracture distance was 4 mm, and empty hole number was 4. In this case, bone union was achieved by inserting screws near the fracture site via revision surgery, which increased stability (Fig. 2). Case 2 was distal femur fracture with Gustilo IIIA in a 73-year-old man; he was run over by a shovel car. Callus formation was good, but resulted in non-union. It showed that plate length was 196 mm, fracture distance was 39 mm, plate span ratio was 196/39 = 5.02, bridge span length was 65 mm, medial fracture distance was 5 mm, and empty hole number was 2. We believe that non-union was influenced by the instability of the fracture segment; therefore, another plate was added on the medial side (Fig. 3).

In case 3, it showed that plate length was 196 mm, fracture distance was 65 mm, plate span ratio was 196/65 = 3.01, bridge span length was 85 mm, medial fracture distance was 0 mm (because of shortening fracture site), and empty hole number was 1. Performing shortening and fixation of up to 5–10 mm was effective for achieving bone union in several comminuted fracture cases (Fig. 4).

## Discussion

During surgery, it is sometimes difficult to properly determine screw position. Thus, the purpose of this study was to examine the appropriate screw position in relative fixation.

The guidelines reported by Gautier et al. suggested that a locking compression plate should be used as a bridging plate in order to achieve relative stability. Additionally, the researchers recommended that the plate length used in simple fractures should be 8–10 times longer than the fracture length, 0–3 empty holes should be left in the surrounding space, the distance space should be ≤ 2 mm, and ≥ 3 screws should be inserted (bicortically) into the proximal and distal bone fragments [14]. For comminuted fractures, the guidelines recommend that the plate length should be at least 2–3 times longer than the fracture length and ≥ 3 empty holes should be left in the surrounding fracture site. Additionally, according to Stoffel et al., the number of empty holes, including the fracture site, should be between one and four in simple fractures, and in comminuted fractures, the screws should be inserted as close to the fracture site as possible [15].

Although screw insertions near the fracture site increases fracture stability, in some cases, this may result in a lack of proper fracture micro-motion, leading to poor callus formation [17]. The “far cortical locking technology” was demonstrated to increase fracture stability and flexibility; however, long-term results have not yet been obtained [18]. Megas et al. reported that fracture site instability prevents bone callus formation, even with good blood supply [19]. In most of our cases, non-union likely resulted from fracture site instability after reduction internal fixation.

Henderson et al. reported no plate length differences between non-union and union groups, which was similar to our results [20]. Elkins reported favorable callus formation with a bridge span length of ≤ 80 mm. Conversely, fracture instability was significantly higher with > 80 mm of bridge span length, which resulted in poor callus formation [21]. Lujan et al. reported that callus formation decreases as the bridge span length increases [22]. Bottland et al. reported that increasing fracture site flexibility promotes initial bone formation, but had no correlation with bridge span length [23]. Additionally, Henderson et al. reported no significant differences in bridge span lengths of 64.4 and 69.8 mm in two respective cases of non-union and union [20]. Our study also showed no significant differences between groups (Tables 2, 3, and 4).

Stoffel et al. advised to reduce the fracture site distance to ≤ 2 mm [15]. In our study, 7 of 26 simple fracture cases had a medial fracture distance of ≥ 2 mm, and non-union was observed in 2 of these 7 (28.5%) cases. Additionally, of the 45 cases with comminuted fractures, 13 cases had a medial fracture distance of ≥ 2 mm, and non-union was observed in 4 of these 13 (30.7%) cases. Bone union was observed in all cases with simple or comminuted fractures with respective medial fracture distances of ≤ 2 mm or ≤ 5 mm. Based on our findings, we concluded that bone fragment distance between fracture fragments is more important than bridge span length of the fracture site and the number of empty holes.

Reducing comminuted bone fragments is difficult when the inner portion of the fracture site also exhibits comminuted fracture. In such cases, shortening the fracture site to approximately 5–10 mm would be acceptable to decrease the distance between the proximal and distal bone fragments.

Regarding the number of empty holes, Stoffel et al. recommended opening 1–2 holes close to the fracture sites in simple fractures and inserting the screws as close as possible to the fracture sites in comminuted fractures [13]. In Henderson et al.’s study involving 70 patients, the average number of empty holes proximal to the fracture site was 0.3 in the non-union group and 1.1 in the bone union group [20]. Additionally, Bottlang et al. observed a 19% bone union failure rate in a cohort of 70 patients and reported that there were significantly more empty holes proximal to the fracture site in the patients with non-union compared to those with successful bone union [23]. In our study, while there were no clear differences in the non-union rates as a result of the number of empty holes, non-union rate tended to increase as the number of empty holes increased in the proximal fragment in some cases. We believe that by decreasing the number of empty holes around the fracture site (i.e., inserting the screw near the fracture site), fracture site stability increased. Even with relative fixation, the more the empty hole, the higher the risk of a non-union, because bone union was achieved during salvage surgery in non-union cases through plate replacement with a long plate or inserting a screw near the fracture site.

Risk factors for non-union and delayed bone union in distal femoral fractures include the presence of open fracture(s), medial bone defects, and comminuted fracture(s) [3, 24]. Compared with the proximal section of the femur or femoral trunk, poor blood supply in the distal femur has also been reported to contribute to a higher risk of non-union [25]. Reports published by various authors are listed in Table 5 [3, 23, 24, 26–31].

**Table 5:** Healing complications of distal femoral fractures treated with locking plates

Study	Number of fractures	Open fractures, %	Non-unions, %	Delayed unions, %	Average time to union, weeks
Schandelmaier et al. [25]	54	19	2	6	13
Fankhauser et al. [26]	30	47	0	3	12
Schutz et al. [27].	52	32	4	12	
Vallier et al. [28]	46	54	9	15	
Kayali et al. [29]	27	26	0		15
Henderson et al. [22]	12		8	8	
Ricci et al. [3]	305	29	14		
Rodriguez et al. [23]	283	18	14		
Harvin et al. [30]	99	29	34		

Study limitations included its retrospective design and the relatively small sample size. A senior medical doctor with > 15 years of experience as an orthopedic surgeon operated on these cases; therefore, a certain surgery quality (reduction of fractures, treatment of soft tissues) was guaranteed. We analyzed several independent variables to determine how they impacted fracture healing; however, it is possible that other variables that were not considered may have contributed to the outcomes. Additionally, although we did not consider bone mineral densities and BMI, there is a possibility that they might have influenced bone union.

## Conclusions

In distal femoral fractures, non-union sometimes may occur, despite improvements in implant and reduction techniques. Well-known risk factors include smoking, bone defects, and comminuted fractures, which were supported by this study’s results. The bone fracture distance on the medial side of the distal femur was related to bone union rather than screw position. These results will be helpful in treating distal femoral fractures with plates.

Based on our findings, we concluded that bone fragment distance between fracture fragments is more important than bridge span length of the fracture site and the number of empty holes. The number of empty holes near the fracture site and the rate of bone healing were not clearly related.

## Data Availability

The datasets used and analyzed during the current study are available from the corresponding author on reasonable request.

## Abbreviations

AO/OTA: Arbeitsgemeinschaft für Osteosynthesefragen/Orthopedic Trauma Association
AP: Antero-posterior
LCP-DF: Locking compression plate for distal femur
